# Deletion of α5 integrin in endothelial cells ameliorates age–associated cognitive decline and increases survival in mice

**DOI:** 10.1002/alz.093892

**Published:** 2025-01-09

**Authors:** Gregory W Hall, Saifudeen Ismael, Gregory J Bix

**Affiliations:** ^1^ Department of Neurosurgery, Clinical Neuroscience Research Center, Tulane University School of Medicine, New Orleans, LA USA; ^2^ Tulane Brain Institute, Tulane University, New Orleans, LA USA

## Abstract

**Background:**

Levels of inflammatory components gradually rise in tissues and blood as we age. This “inflammageing” process is often debilitating and even fatal. Cognitive impairment is one example of inflammageing’s incapacitating nature. Excessive permeation of inflammatory markers in the brain gradually erodes the blood‐brain barrier (BBB) over time. Consequently, enhanced cerebral inflammatory processes contribute to neurological senescence. Neurological inflammageing may be partly due to a5 integrin, a receptor found primarily in brain endothelial cells during brain development, cerebrovascular injury (e.g. stroke), neuroinflammation and neurodegenerative disease (e.g. ALS). We hypothesize that deletion of a5 integrin in endothelial cells helps maintain BBB integrity and leads to improved cognitive performance with age. In the past we have shown that endothelial cell selective a5 knockout (a5‐EC‐KO) mice are more resilient to ischemic stroke. Enhanced stroke recovery in a5‐EC‐KO mice is due to less a5‐related BBB deterioration and subsequently less brain edema and infiltration of peripheral inflammatory cells. Therefore, we used aged a5‐EC‐KO and wild type (WT) litter mate mice to investigate how a5 integrin influences cognitive performance and survival probability over time.

**Methods:**

25‐32‐month‐old male and female a5‐EC‐KO mice and aged‐matched WT mice were used in the study. Cognitive function was analyzed using Y‐maze and open field. Additionally, Kaplan Meier curves were used to assess probability of survival.

**Results:**

For Y maze, results indicate a trend towards greater alteration rate and total alteration for a5‐EC‐KO mice. Open field test shows greater center time and less peripheral time for a5‐EC‐KO. WT and a5‐EC‐KO mice have nearly identical mean speed. Additionally, Kaplan‐Meier curves indicate a greater probability of survival for a5‐EC‐KO mice between the ages of 26 to 30 months. Interestingly, 26‐30 months is the average life expectancy of a lab mouse.

**Conclusions:**

Observations from Y maze and open field tests suggest that a5‐EC‐KO mice have improved spatial memory and less anxiety behavior when compared to aged‐matched WT mice. Moreover, the Kaplan‐Meier curves depict a greater probability of survival for aged a5‐EC‐KO mice. Thus, a5 integrin expression may significantly influence age‐related cognitive decline. Preliminary data suggests that inhibition of a5 integrin may improve symptoms of age‐associated cognitive impairment.

